# Visceral Leishmaniasis diagnosis: a rapid test is a must at the hospital bedside

**DOI:** 10.6061/clinics/2020/e2036

**Published:** 2020-06-16

**Authors:** Lucia Maria Almeida Braz, Roozbeh Tahmasebi, Philip Michael Hefford, José Angelo Lauletta Lindoso

**Affiliations:** I Instituto de Medicina Tropical, Faculdade de Medicina FMUSP, Universidade de Sao Paulo, Sao Paulo, SP, BR.; II Escola Politecnica, Universidade de Sao Paulo, Sao Paulo, SP, BR.; III University Hospitals Plymouth NHS Trust, Plymouth, England.; IV Instituto de Infectologia Emilio Ribas, Sao Paulo, SP, BR.

At the time of the widespread availability of rapid diagnostic tests for SARS-CoV-2—the causative virus of the COVID-19 pandemic—from drugstores throughout Brazil, there is a distinct lack of use of rapid diagnostic tests for visceral leishmaniasis (VL) at the bedsides of hospitalized patients. These tests are mainly distributed by the Ministério da Saúde do Brazil-MS (Ministry of Health) only to the Laboratórios Centrais de Saúde Publica - LACENs (Central Public Health Laboratories) and are predominantly provided to public hospitals.

VL is the most severe form of leishmaniasis and it causes high morbidity and mortality in developing countries (1,2). *Leishmania infantum chagasi* is responsible for VL in the New World, with typical clinical signs and symptoms of splenomegaly, hepatomegaly, and fever (3). VL can be life-threatening, and because 90% cases of VL occur in Brazil, reliable and rapid diagnosis of VL is required (4).

As stated by the MS, VL case confirmation is based on clinical suspicion and positive laboratory diagnosis via either parasitological tests (PTs), which are dependent on invasive procedures such as bone marrow aspiration or biopsy, or serological tests such as indirect immunofluorescence (IFI) or immunochromatographic tests (ITs) using rK39 recombinant antigens (5). The serological tests IFI and IT-rK39 have the advantage of being minimally invasive and they can be performed in large numbers (6).

However, IFI requires a fluorescence microscope and is time consuming. The procedure of IT-rK39 takes only 10-15 minutes and requires only 10-20 µL of the peripheral blood. It is a rapid and low-cost bedside test. The rK39 dipstick used for ITs is the product of a gene cloned from the *Leishmania* genus containing a 39-amino acid repeat conserved among viscerotropic *Leishmania* species (7).

The main brands of IT-rK39 that were previously provided by the Brazilian public health system consisted of Kalazar Detect™ (InBios International, Seattle, WA, USA), IT LEISH® (BIO-RAD Laboratories Inc., France), and OnSite™ Leishmania IgG/IgM Combo test (CTK Biotech, USA), which have now been replaced with the LSH Ab ECO test (Eco Diagnóstica, Nova Lima, MG, Brasil). Kalazar Detect™ was the first rapid test for VL diagnosis that was adopted by the Brazilian public health system in 2009. It has a sensitivity and specificity of 88.1% and 90.6%, respectively.

In 2015, IT LEISH® replaced the Kalazar Detect™ and showed an improved sensitivity and specificity of 93% and 97%, respectively (8). However, these IT-rK39 tests would usually present a lower accuracy when tested in patients coinfected with HIV (9,10).

In 2017, the OnSite™ *Leishmania* IgG/IgM Combo test replaced the IT LEISH® (8). Today, MS recommends using a new brand, the LSH Ab ECO test, a qualitative immunoassay for the detection of antibodies (rK39) against VL in human serum (11). The specificity for this test is equal to 100% (95% CI 0.93-1), indicating that it has high specificity for the rK39 protein. The sensitivity presented by the LSH Ab ECO is 92% (95% CI 0.82-0.97) (11).

LSH Ab ECO test was declared by the Agência Nacional de Vigilância Sanitária-ANVISA (National Health Surveillance Agency) as a criterion for the laboratory confirmation of suspected cases of the disease. Therefore, suspicious patients, including those presenting with clinical signs compatible with disease and those coming from a region with known occurrence of transmission, alongside a positive rapid test, can be considered confirmed cases of VL based on clinical laboratory criteria.

The LSH Ab ECO test has technical specifications and execution methodology similar to those of the brands used before. According to the manufacturer, LSH Ab ECO, a lateral flow chromatographic immunoassay used to detect class G immunoglobulin for *Leishmania donovani*, uses recombinant antigens in the test line and chicken anti-protein A in the control line. It is easy to use and interpret. In accordance with the manufacturer’s instructions and technical orientation from SDP/IOM/FUNED n°001/2019 (12), the procedure of the test is as follows: add 20 μL of serum/plasma or 1 drop of blood (10 µL) to the test strip pad, below the arrows. If serum, plasma, or blood is applied to the test strip horizontally on a flat surface, take the strip by the green label and place it vertically, with the arrow pointing downwards, in a test tube or microwell containing 2-3 drops (150 μL) of the diluent buffer. If serum, plasma, or blood is applied to the test strip vertically, add 2-3 drops (150 μL) of the diluent buffer to the base of the microwell or test tube and read the test result after 10 minutes.

It is important to highlight that this IT for rK39 is produced by a Brazilian biotechnology industry located in the state of Minas Gerais, Brazil, as the previously used brands were produced by industries situated outside of Brazil. This is an important achievement for the Brazilian health system with regard to VL diagnosis.

In summary, the test is suitable for use at the bedside, requires a minimal amount (10 μL) of peripheral blood, with no need of special equipment, and is simple to perform and read, with the results being available in 10 minutes. However, this simple dipstick test for rK39, distributed by the Ministério da Saúde do Brazil to public laboratories, is not available in some public hospitals, including Hospital das Clinicas da Faculdade de Medicina da Universidade de São Paulo (HCFMUSP). Rapid test-rK39 is not even offered by the majority of private laboratories and private hospitals for VL diagnosis. Even if it is provided, the turnaround time between sending the sample and receiving results is typically a minimum of 24 hours; thus, it can hardly be deemed as a ‘rapid test’ with any conviction.

It is time to change the narrative and alter the distributive flowchart of this test. It is necessary to use the rK39 IT at the bedside of suspected VL patients across hospitals to the greatest effect. Why not employ the technical skills of a team who usually attend patient needs, such as nurses and nursing technicians, thereby ensuring that the rK39 IT truly does indeed become a rapid diagnostic bedside test? VL can be lethal and patients simply cannot afford to wait for diagnoses/treatments.

## AUTHOR CONTRIBUTIONS

Braz LMA and Lindoso JAL designed the study, drafted and reviewed the manuscript. Tahmasebi R and Hefford PM reviewed the manuscript, also for English language. All of the authors have read and approved the content of the manuscript.
